# Prediction of peak oxygen uptake in children using submaximal ratings of perceived exertion during treadmill exercise

**DOI:** 10.1007/s00421-016-3377-z

**Published:** 2016-04-22

**Authors:** Danielle Lambrick, Haley Bertelsen, Roger Eston, Lee Stoner, James Faulkner

**Affiliations:** Faculty of Health Sciences, University of Southampton, Highfield Campus, Southampton, SO17 1BJ UK; School of Sport and Exercise, Massey University, Wellington, New Zealand; Alliance for Research in Exercise, Nutrition and Activity, School of Health Sciences, University of South Australia, Adelaide, Australia; Faculty of Business, Law and Sport, University of Winchester, Winchester, UK

**Keywords:** $\dot{V}{\text{O}}_{{2{\text{peak}}}}$, CPET, Paediatrics, RPE, Treadmill

## Abstract

**Purpose:**

This study assessed the utility of the Children’s Effort Rating Table (CERT) and the Eston–Parfitt (EP) Scale in estimating peak oxygen uptake ($\dot{V}{\text{O}}_{{2{\text{peak}}}}$) in children, during cardiopulmonary exercise testing (CPET) on a treadmill.

**Methods:**

Fifty healthy children (*n* = 21 boys; 9.4 ± 0.9 years) completed a continuous, incremental protocol until the attainment of $\dot{V}{\text{O}}_{{2{\text{peak}}}}$. Oxygen uptake ($\dot{V}{\text{O}}_{2}$) was measured continuously, and ratings of perceived exertion (RPE) were estimated at the end of each exercise stage using the CERT and the EP Scale. Ratings up to- and including RPE 5 and 7, from both the CERT (CERT 5, CERT 7) and EP Scale (EP 5, EP 7), were linearly regressed against the corresponding $\dot{V}{\text{O}}_{2}$, to both maximal RPE (CERT 10, EP 10) and terminal RPE (CERT 9, EP 9).

**Results:**

There were no differences between measured- and predicted $\dot{V}{\text{O}}_{{2{\text{peak}}}}$ from CERT 5, CERT 7, EP 5 and EP 7 when extrapolated to either CERT 9 or EP 9 (*P* > 0.05). Pearson’s correlations of *r* = 0.64–0.86 were observed between measured- and predicted $\dot{V}{\text{O}}_{{2{\text{peak}}}}$, for all perceptual ranges investigated. However, only EP 7 provided a small difference when considering the standard error of estimate, suggesting that the prediction of $\dot{V}{\text{O}}_{{2{\text{peak}}}}$ from EP 7 would be within 10 % of measured $\dot{V}{\text{O}}_{{2{\text{peak}}}}$.

**Conclusions:**

Although robust estimates of $\dot{V}{\text{O}}_{{2{\text{peak}}}}$ may be elicited using both the CERT and EP Scale during a single CPET with children, the most accurate estimates of $\dot{V}{\text{O}}_{{2{\text{peak}}}}$ occur when extrapolating from EP 7.

## Introduction

Maximal- or peak oxygen uptake ($\dot{V}{\text{O}}_{{2{ \text{max} }}}$ or $\dot{V}{\text{O}}_{{2{\text{peak}}}}$) is the gold-standard test of cardiorespiratory fitness in both adults and children (Armstrong et al. 1996; Howley et al. 1995; Wilmore and Costill 2004) and is routinely assessed during cardiopulmonary exercise testing (CPET). However, engaging certain non-athletic or patient populations in maximal exercise testing may not be suitable, safe or practical, and if undertaken, such individuals may develop various exercise-induced physiological signs or complaints that warrant the termination of the test prior to maximal values being attained (Jankowski et al. 2015; Noonan and Dean 2000; Takken et al. 2009). In recent years, the Borg 6–20 ratings of perceived exertion (RPE) scale has been shown to be a valid, reliable and sensitive tool to predict $\dot{V}{\text{O}}_{{2{ \text{max} }}}$ in healthy active- and sedentary individuals, and in certain clinical populations (Coquart et al. 2014; Faulkner and Eston 2007). Its utility has been shown during perceptually regulated exercise tests—an active process in which the individual is asked to self-regulate and maintain an exercise intensity corresponding to a prescribed RPE (Eston et al. 2005, 2006, 2008, 2012; Evans et al. 2013; Faulkner et al. 2007; Morris et al. 2010)—and during CPET—where an individual is typically asked to passively estimate his or her perception of exertion during each stage of the test (Al-Rahamneh and Eston 2011; Al-Rahamneh et al. 2011; Coquart et al. 2009, 2015; Faulkner and Eston 2007; Lambrick et al. 2009). However, to the best of our knowledge, research has not assessed the predictive utility of submaximal RPE during a single CPET to obtain estimates of $\dot{V}{\text{O}}_{{2{\text{peak}}}}$ in children. This may prove important when assessing the cardiorespiratory fitness of children who are in the pre- or post-operative stage, or who are considered to have clinical co-morbidities (obese), where a CPET to maximal exertion may not be suitable. In the interests of safety, assessing such predictive methodologies on a healthy paediatric population initially is considered prudent.

Moderate to strong linear relationships have been reported between the RPE and both oxygen uptake ($\dot{V}{\text{O}}_{2}$) and heart rate (HR) in children (Roemmich et al. 2006; Utter et al. 2002). Hence, a number of paediatric-specific, *linear*, perceived exertion scales have been validated for the purposes of estimating or guiding exercise intensity. One such tool is the Children’s Effort Rating Table (CERT) (Williams et al. 1994) which has demonstrated validity and reliability during cycling (Eston et al. 1994; Lamb 1995, 1996; Leung et al. 2002), stepping (Yelling et al. 2002) and treadmill exercise (Marinov et al. 2008; Roemmich et al. 2006). The CERT uses a 1–10 numerical range with associated verbal anchors, ranging from ‘Very Very Easy’ (CERT 1) to ‘So Hard I’m Going to Stop’ (CERT 10), that are deemed familiar to a child, but, it does not contain any pictorial descriptors. There is a strong rationale, however, for the use of a *curvilinear* RPE scale with children, as it has previously been shown that a curvilinear design [Eston–Parfitt (EP) Scale] (Eston et al. 2009) may better reflect the nature of effort perception in relation to exercise of progressively increasing intensity in children, during both cycle ergometry (Eston et al. 2009) and treadmill (Lambrick et al. 2011) exercise. The EP Scale uses a similar, 0–10 numerical range and verbal anchors abridged from the CERT, and it depicts a character at various stages of exertion on a concave slope with a progressively increasing gradient at the higher intensities. Accordingly, the distance between each numbered increment on the horizontal axis (0–10) is increasingly reduced in relation to its antecedent. To date, it has not been ascertained whether the CERT or EP Scale can accurately predict $\dot{V}{\text{O}}_{{2{\text{peak}}}}$ in children.

With adults, two extrapolation end points are frequently utilised when assessing the predictive utility of the RPE during submaximal exercise procedures. The first is the theoretical maximal RPE, dictated by the highest value on the perceptual scale being employed (e.g., RPE 20, when employing the Borg 6–20 Scale). The second is the peak terminal RPE, defined as the highest value typically reported at the end of an exhaustive exercise test (e.g., RPE 19, when employing the Borg 6–20 Scale) (Faulkner et al. 2007; Lambrick et al. 2009). Both are considered suitable indicators of maximal exertion within the adult literature; however, there is no consensus as to which proves more accurate when assessing the predictive utility of the RPE. Like adults, children have also been shown to report terminal RPE values that are lower than theoretical maximal RPE at the termination of an exhaustive exercise test (Eston et al. 2009; Lambrick et al. 2011). Eston et al. (2009), for example, demonstrated that children reported average, terminal RPE values of 9.4 ± 1.1 when using the EP Scale (with a theoretical maximum of 10). As such, it would be judicious to utilise two extrapolation end points (CERT 9 and CERT 10, EP 9 and EP 10) when assessing the predictive utility of the CERT and EP Scale with children.

The purpose of this study was to assess (1) whether submaximal RPE, obtained during a single CPET on a treadmill, can accurately predict measured $\dot{V}{\text{O}}_{{2{\text{peak}}}}$ in children, and (2) whether the accuracy of the $\dot{V}{\text{O}}_{{2{\text{peak}}}}$ predictions are influenced by the perceptual scale employed (CERT vs. EP Scale). It was hypothesised that the EP Scale would more accurately predict $\dot{V}{\text{O}}_{{2{\text{peak}}}}$ and that an extrapolation endpoint of RPE 9 would be more accurate than an RPE 10.

## Methods

### Participants

Fifty healthy children (*n* = 21 boys; age: 9.4 ± 0.9 y; height: 1.42 ± 0.09 m; body mass: 41.6 ± 13.0 kg; body mass index: 20.3 ± 4.5 kg m^2^; body fat: 26.1 ± 8.1 %) volunteered for the study. All participants were asymptomatic of any pre-existing injury or illness, as determined by a health history questionnaire, and informed, written parental consent and child assent were obtained prior to participation. This research was conducted in agreement with the guidelines and testing policies of the Institutional Human Ethics Committee, in conjunction with the Declaration of Helsinki.

### Procedures

Each participant performed a single graded exercise test (GXT) on a treadmill (True 825, Fitness Technologies, St. Louis, USA), in a thermoneutral laboratory environment (temperature: 22.2 ± 1.1 °C; humidity: 33.4 ± 4.4 %; pressure: 1006 ± 9.0 Pa). Prior to the GXT, participants were familiarised to the treadmill and other equipment to be used in the study. Children practiced running on the treadmill and performed safe stopping techniques until they were comfortable with the equipment. They were introduced to two RPE scales (CERT and EP Scale) and provided with both a verbal explanation (employing anchoring techniques) and a practical demonstration (whilst practicing their running on the treadmill) on how to use these scales, prior to the start of the GXT. They were subsequently provided with a rest period, during which the GXT protocol was explained to them, any questions were answered, and their HR returned to baseline levels. The treadmill gradient was set at 1 % throughout the GXT (Jones and Doust 1996). Respiratory variables including $\dot{V}{\text{O}}_{2}$, minute ventilation ($\dot{V}_{\text{E}}$) and respiratory exchange ratio (RER) were recorded using a breath-by-breath automatic gas exchange system (Sensormedics Corporation, Yorba Linda, CA, USA). Children wore a paediatric wireless chest strap telemetry system to monitor HR (Polar Electro T31, Kempele, Finland). All physiological variables were recorded continuously throughout the exercise protocols, but were concealed from participants. RPE scales remained visible to participants whilst exercising, and RPE was assessed during the final 15 s of each stage of the GXT.

### Graded exercise test

The GXT consisted of a continuous, incremental protocol that commenced at a speed of 7 km h^−1^ and increased by 0.5 km h^−1^ every minute until the attainment of $\dot{V}{\text{O}}_{{2{\text{peak}}}}$, as determined by volitional exhaustion. Following a 15-min passive recovery, and confirmation that HR had returned to within 10 % of baseline resting values, a ‘verification test’, wherein children ran at 105 % of the peak treadmill speed achieved from the preceding maximal GXT until volitional exhaustion, was used to validate $\dot{V}{\text{O}}_{{2{\text{peak}}}}$ (Barker et al. 2011).

### Data analysis

The final 10 s of $\dot{V}{\text{O}}_{2}$ data, from each stage of the GXT, were averaged and used in subsequent analyses. A paired samples *t* test was used to compare the difference in the terminal RPE between the CERT and the EP Scale. Similarly, a paired samples *t* test was used to compare the submaximal $\dot{V}{\text{O}}_{2}$ recorded at CERT 5 against that recorded at EP 5, as well as between CERT 7 and EP 7. Simple linear regression analysis was employed on the submaximal RPE and $\dot{V}{\text{O}}_{2}$ values up to- and including both an RPE of 5 and 7 on CERT, extrapolated to both the theoretical maximal- (CERT 10) and peak terminal RPE (CERT 9), for each participant. For the EP Scale, due to the curvilinear nature of the RPE response (Eston et al. 2009; Lambrick et al. 2011), the corresponding submaximal RPE and $\dot{V}{\text{O}}_{2}$ values (≤RPE of 5 and 7) for each participant were log-transformed and then linearly regressed to obtain an appropriate *b* coefficient and constant. Thereafter, logged variables were transformed into their inverse to obtain a prediction of $\dot{V}{\text{O}}_{{2{\text{peak}}}}$, when extrapolated to both EP 10 and EP 9. RPE 5 and RPE 7 were chosen as points for extrapolation as they correspond to moderate- and high exercise intensities, respectively, in children. In adults, accurate predictions of $\dot{V}{\text{O}}_{{2{ \text{max} }}}$ have been elicited when extrapolating from both a moderate- (e.g., RPE 13, when employing the Borg 6–20 Scale) and high- (e.g., RPE 15, when employing the Borg 6–20 Scale) exercise intensity (Faulkner and Eston 2007; Lambrick et al. 2009). A one-way analysis of variance (ANOVA) was used to compare the predicted $\dot{V}{\text{O}}_{{2{\text{peak}}}}$, which had been extrapolated from an RPE of 5 to both RPE 10 and RPE 9, using both CERT and the EP Scale, to measured $\dot{V}{\text{O}}_{{2{\text{peak}}}}$. An identical analysis was performed when extrapolating from RPE 7. Pearson’s correlations (*r*) were used to assess the strength of the relationship between the measured- and predicted $\dot{V}{\text{O}}_{{2{\text{peak}}}}$ (from RPE 5 and 7, to RPE 9 and 10, for both CERT and the EP Scale). In general, *r* values above 0.75 are considered to indicate excellent agreement, and values between 0.4 and 0.74 are considered a fair to good agreement. The uniformity of error was assessed by visual analysis of regression plots, and the standard error of estimate (SEE) was derived from the regression analysis to provide an estimation of random error (Hopkins 2000). In addition, the SEE was divided by the SD of the predicted $\dot{V}{\text{O}}_{{2{\text{peak}}}}$ to provide a standardised indicator of error, whereby <0.20 is considered a trivial difference, 0.2-0.6 small, 0.6–1.2 moderate, 1.2–2.0 large, and >2.0 very large difference. The relative standard error (RSE) was also calculated by expressing SEE relative to the mean of the criterion (measured $\dot{V}{\text{O}}_{{2{\text{peak}}}}$). All data were analysed using the statistical package SPSS for Windows (PC software, Version 21.0).

## Results

### GXT to $\dot{V}{\text{O}}_{{2{\text{peak}}}}$

The peak physiological values from the GXT were: (mean ± SD) HR: 203 ± 7 b min^−1^; $\dot{V}{\text{O}}_{2}$: 55.2 ± 10.8 ml kg^−1^ min^−1^; RER: 1.04 ± 0.1; $\dot{V}_{\text{E}}$: 73.3 ± 16.2 L min^−1^. Peak speed was 10.9 ± 1.2 km h^−1^. Maximal perceptual values for the EP Scale and CERT were identical: 9.6 ± 0.7 (*P* > 0.05). The $\dot{V}{\text{O}}_{2}$ values recorded at CERT 5 (43.1 ± 8.4 ml kg^−1^ min^−1^) were lower than those at EP 5 (45.5 ± 8.5 ml kg^−1^ min^−1^; *t*_(48)_ = −5.05, *P* < 0.001). There were no differences in the $\dot{V}{\text{O}}_{2}$ values recorded at CERT 7 (49.3 ± 9.7 ml kg^−1^ min^−1^) and EP 7 (49.8 ± 9.7 ml kg^−1^ min^−1^; *P* > 0.05). The submaximal $\dot{V}{\text{O}}_{2}$ values equated to $\dot{V}{\text{O}}_{{2{\text{peak}}}}$ percentages of 78 ± 10, 83 ± 10, 90 ± 11, and 91 ± 10 %, for CERT 5, EP 5, CERT 7 and EP 7, respectively.

### Predicting $\dot{V}{\text{O}}_{{2{\text{peak}}}}$ from RPE 5

Significant differences were observed between measured- and predicted (CERT 5, EP 5) $\dot{V}{\text{O}}_{{2{\text{peak}}}}$ (*F*_(4,227)_ = 5.41, *P* < 0.001; Table 1). Post hoc analysis demonstrated that both CERT 5 (95 % CI 59.5–69.8 ml kg^−1^ min^−1^) and EP 5 (95 % CI 52.7–63.1 ml kg^−1^ min^−1^) significantly overestimated measured $\dot{V}{\text{O}}_{{2{\text{peak}}}}$ when extrapolated to RPE 10 (*P* < 0.01). However, there were no differences between measured- and predicted $\dot{V}{\text{O}}_{{2{\text{peak}}}}$ when extrapolated to RPE 9 (*P* > 0.05) from either CERT 5 (95 % CI 56.1–65.9 ml kg^−1^ min^−1^) or EP 5 (95 % CI 51.7–59.4 ml kg^−1^ min^−1^).

**Table 1:** Comparison between measured- and predicted peak oxygen uptake ($\dot{V}{\text{O}}_{{2{\text{peak}}}}$) values

		*N* ^a^	$\dot{V}{\text{O}}_{{2{\text{peak}}}}$ (ml kg^−1^ min^−1^)				Mean diff.	*r*	SEE absol.	SEE stand.	RSE %	Likelihood
			Actual		Predicted							
			*X*	(SD)	*X*	(SD)						
RPE 10												
CERT 5		41	54.7	(11.2)	64.7	(16.4)	10.1	0.72	11.4	1.02	20.8	Mod.
CERT 7		50	55.2	(10.8)	59.7	(13.8)	4.86	0.68	10.1	0.94	18.4	Mod.
EP 5		48	54.9	(10.8)	57.9	(14.8)	3.34	0.64	11.4	1.05	20.8	Mod.
EP 7		50	55.2	(10.8)	56.1	(12.1)	1.29	0.84	6.63	0.61	12.0	Mod.
RPE 9												
CERT 5		41	54.7	(11.2)	61.0	(15.5)	6.35	0.74	10.5	0.93	19.1	Mod.
CERT 7		50	55.2	(10.8)	56.6	(11.9)	1.29	0.70	8.49	0.78	15.3	Mod.
EP 5		48	54.9	(10.8)	55.6	(13.2)	0.71	0.69	9.61	0.89	17.5	Mod.
EP 7		50	55.2	(10.8)	54.1	(11.2)	−1.13	0.86	5.64	0.52	10.2	Small

*r*, Pearson correlation coefficients; SEE (absol.), standard error of estimate in absolute terms; SEE (stand.), standard error of estimate relative to the standard deviation of the criterion; RSE, relative standard error; likelihood, where an SEE (stand.) of <0.20 is considered a trivial difference, 0.2–0.6 small, 0.6–1.2 moderate, 1.2–2.0 large, and >2.0 very large difference

^a^NB. Differing sample sizes result when insufficient perceptual data have been obtained to allow for predictions using linear regression analysis

### Predicting $\dot{V}{\text{O}}_{{2{\text{peak}}}}$ from RPE 7

There were no significant differences between measured- and predicted (CERT 7, EP 7) $\dot{V}{\text{O}}_{{2{\text{peak}}}}$ when extrapolated to either RPE 9 or RPE 10 (*F*_(4,248)_ = 2.26, *P* > 0.05; Table 1). Both CERT 7 (95 % CI 53.2–60.0 ml kg^−1^ min^−1^) and EP 7 (95 % CI 50.9–57.3 ml kg^−1^ min^−1^) provided a closer estimate of the actual $\dot{V}{\text{O}}_{{2{\text{peak}}}}$ when extrapolated to an RPE 9 than when either were extrapolated to an RPE 10 (95 % CI 55.7–63.6 ml kg^−1^ min^−1^ for CERT 7; 95 % CI 53.6–60.4 ml kg^−1^ min^−1^ for EP 7).

### Pearson’s correlations and SEE

**Fig. 1:**
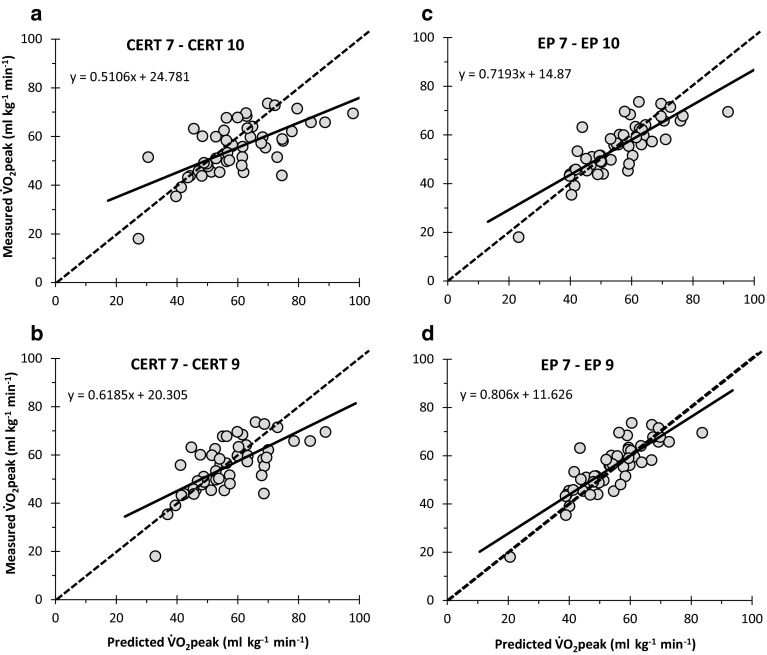
Correlation plots between measured peak oxygen uptake ($\dot{V}{\text{O}}_{{2{\text{peak}}}}$) and $\dot{V}{\text{O}}_{{2{\text{peak}}}}$ predicted from **a** CERT 7 to CERT 10, **b** CERT 7 to CERT 9, **c** EP 7 to EP 10, and **d** EP 7 to EP 9. Line of best fit (*solid line*) and perfect agreement (*dashed line*) are shown

Pearson’s correlations (*r*) between measured- and predicted $\dot{V}{\text{O}}_{{2{\text{peak}}}}$ were higher for predictions of $\dot{V}{\text{O}}_{{2{\text{peak}}}}$ using the EP Scale compared to CERT, and when extrapolated to RPE 9 compared to RPE 10 (Table 1). With regard to the SEE, these were superior for RPE 7 than RPE 5 for both the EP Scale and CERT. Extrapolation to RPE 9 was also shown to be superior than RPE 10, while extrapolation from EP 7 to RPE 9 was the only procedure which demonstrated a small difference to the criterion measure (Table 1; Fig. 1).

## Discussion

This is the first study to show that the relationship between submaximal $\dot{V}{\text{O}}_{2}$ and RPE during a single CPET can be used to predict measured $\dot{V}{\text{O}}_{{2{\text{peak}}}}$ in children, aged 8–10 years. Of the two perceptual scales employed in this study, more accurate predictions, in conjunction with smaller differences in the SEE, were obtained when using the EP Scale as opposed to CERT. This indicates that the EP Scale may be a more appropriate perceptual scale for use with young children. In accordance with previous findings from the adult literature (Faulkner and Eston 2007), more accurate estimates of $\dot{V}{\text{O}}_{{2{\text{peak}}}}$ were identified when extrapolating from a higher perceptual range (RPE 7), due to the lesser potential for error in prediction when using the extrapolation method, and when extrapolated to the terminal RPE (RPE 9).

Although the relationship between RPE and $\dot{V}{\text{O}}_{2}$ is generally presumed to be linear in both adults and children, it has been shown previously that a curvilinear RPE scale may better reflect the nature of perceived exertion responses in young children during exercise of increasing intensity (Lambrick et al. 2011). In this study, the EP Scale was generally more accurate at predicting $\dot{V}{\text{O}}_{{2{\text{peak}}}}$ in young children than CERT. This was true for predictions from both a lower perceptual range ($\dot{V}{\text{O}}_{{2{\text{peak}}}}$ overestimated by 11 vs. 1 % for CERT 5 and EP 5, respectively) and a higher perceptual range ($\dot{V}{\text{O}}_{{2{\text{peak}}}}$ overestimated by 3 % vs. underestimated by 2 % for CERT 7 and EP 7, respectively), when extrapolated to RPE 9. As poorer predictions of $\dot{V}{\text{O}}_{{2{\text{peak}}}}$ were also obtained when extrapolating to an RPE 10 (Table 1), one could argue that the use of the EP Scale, and the extrapolation of data to terminal RPE (RPE 9), may, as shown for adults (Eston et al. 2007; St Clair Gibson et al. 1999), be more ecologically valid for children. It is interesting that both adults and children tend to report a terminal RPE at the end of an exhaustive exercise test that is lower than the theoretical maximal RPE for any given RPE scale used. It has been postulated in the adult literature that the brain tightly regulates power output to ensure that an exercise task is completed successfully, within the body’s biomechanical and metabolic limits (St Clair Gibson and Noakes 2004). By employing a physiological and perceptual reserve capacity (i.e., RPE 9) at the point of volitional exhaustion, it is suggested that an individual would avoid ‘catastrophic physiological failure’ (St Clair Gibson and Noakes 2004). Although such a mechanism remains speculative, it offers some explanation as to the differences often observed between terminal and theoretical maximal RPEs reported at the end of an exhaustive exercise test.

Based upon the above findings, it is plausible that a health professional could obtain statistically accurate predictions of $\dot{V}{\text{O}}_{{2{\text{peak}}}}$ in 8–10 years old children from a single CPET, particularly if using EP 7 (Table 1). In theory, therefore, this could be beneficial within clinical practice whereby CPET is increasingly used in perioperative care (Arena and Sietsema 2011). In general, Pearson’s correlations demonstrated fair to good agreement between measured- and predicted $\dot{V}{\text{O}}_{{2{\text{peak}}}}$ for all perceptual ranges investigated (*r* = 0.64–0.86), in keeping with previous research with adults (Faulkner and Eston 2007). Typically, the EP Scale provided stronger Pearson’s correlations at the higher perceptual range (RPE 7), whereas CERT proved stronger at the lower perceptual range (RPE 5). When considering the SEE, however, only EP 7 provided a small difference and low bias between measured- and predicted $\dot{V}{\text{O}}_{{2{\text{peak}}}}$ (Table 1; Fig. 1). The RSE also suggested that the prediction of $\dot{V}{\text{O}}_{{2{\text{peak}}}}$ from EP 7 would be within 10 % of the true value. In addition, it should be noted that when using CERT, there was a perceptible underestimation of $\dot{V}{\text{O}}_{{2{\text{peak}}}}$ with individuals of lower fitness, and an overestimation of $\dot{V}{\text{O}}_{{2{\text{peak}}}}$ in individuals of greater fitness, which was not observed with the EP scale (Fig. 1). The SEE and RSE, therefore, provide further evidence that the EP scale is an appropriate perceptual scale for use with children during CPET.

## Conclusion

Acceptable predictions of $\dot{V}{\text{O}}_{{2{\text{peak}}}}$ can be obtained when using both the CERT and EP Scale, during a single CPET, with children. Overall, the EP Scale proves more accurate than CERT for predicting $\dot{V}{\text{O}}_{{2{\text{peak}}}}$, providing further support to the use of a curvilinear RPE scale for obtaining accurate estimates of perceived exertion in children. This is further evident when considering the mean data, Pearson’s correlations and SEE. More accurate predictions of $\dot{V}{\text{O}}_{{2{\text{peak}}}}$ are attained when extrapolating from higher perceptual ranges (RPE 7), and when extrapolated to the terminal RPE (RPE 9). As previous research involving healthy, active and sedentary adult populations has demonstrated the utility of trial familiarisation in improving the predictive accuracy of the RPE (Coquart et al. 2014), future research could investigate whether predictions of $\dot{V}{\text{O}}_{{2{\text{peak}}}}$, obtained during CPET, may be improved with practice in children.

## Abbreviations

ANOVA: Analysis of variance
CERT: Children’s Effort Rating Table
CPET: Cardiopulmonary exercise testing
EP Scale: Eston–Parfitt Scale
GXT: Graded-exercise test
HR: Heart rate
RER: Respiratory exchange ratio
RPE: Ratings of perceived exertion
SEE: Standard error of estimate
$\dot{V}{\text{O}}_{{2({\text{max/peak)}}}}$: (Maximal/peak) oxygen uptake
$$\dot{V}_{\text{E}}$$: Minute ventilation
